# Supplementary material for: Dynamic transcriptomic profiles of zebrafish gills in response to zinc supplementation
Source: BMC Genomics. 2010 Oct 11;11:553. doi: 10.1186/1471-2164-11-553 (PMC3091702; doi:10.1186/1471-2164-11-553)
Supplement: Additional file 2 — Interactive Direct Interaction Network representing the molecular interactions between zinc, copper, iron, calcium and proteins encoded by transcripts changed by zinc supplementation. Mini web-site containing index.html and hyperlinked pages in subdirectory describing a Direct Interaction Network automatically generated based on curated interactions contained within the proprietary PathwayArchitect database. Ovals represent proteins and the circles symbolize metal ions. Objects are coloured by their abundance in zebrafish at the time-point they were significantly different from the control is a scale from -4 fold (dark green) to +4 fold (dark red). Where significant differences were found at more than one time-point, the colour overlay shows expression at the first instance. Dark blue squares denote 'binding', and light blue squares 'expression'; green squares stand for 'regulation', green diamonds for 'metabolism', and green circles for 'promoter binding'. Arrow heads indicate directionality of the interaction where annotated. All nodes and edges can be further interrogated by selecting the relative area of the image. [file 1471-2164-11-553-S2.zip › PathwayArchitect Zn xs DIN/153725.html]

# PROTEIN: APP

|  |  |
| --- | --- |
| Name | APP |
| Type | PROTEIN |
| Description | amyloid beta (A4) precursor protein (peptidase nexin-II, Alzheimer disease) |
| Note | This gene encodes a cell surface receptor and transmembrane precursor protein that is cleaved by secretases to form a number of peptides. Some of these peptides are secreted and can bind to the acetyltransferase complex APBB1/TIP60 to promote transcriptional activation, while others form the protein basis of the amyloid plaques found in the brains of patients with Alzheimer disease. Mutations in this gene have been implicated in autosomal dominant Alzheimer disease and cerebroarterial amyloidosis (cerebral amyloid angiopathy). Multiple transcript variants encoding several different isoforms have been found for this gene. |
| Alias | beta-amyloid peptide |
|  | protease nexin II |
|  | amyloid beta A4 protein |
|  | amyloid beta (A4) precursor protein (protease nexin-II, Alzheimer disease) |
|  | appican |
|  | E030013M08Rik |
|  | AD1 |
|  | cerebral vascular amyloid peptide |
|  | Cvap |
|  | Adap |
|  | A4 amyloid protein |
|  | APP |
|  | ABETA |
|  | betaAPP |
|  | App |
|  | CTFgamma |
|  | Alzheimer's disease amyloid protein homolog |
|  | APPI |
|  | ABPP |
|  | AL024401 |
|  | AG |
|  | amyloid-beta protein |
|  | protease nexin-II |
|  | CVAP |
|  | PN2 |
|  | amyloid |
|  | AAA |
|  | Amyloidogenic glycoprotein |
|  | Abeta |


---

|  |  |
| --- | --- |
| GO Component | axon |
|  | Golgi apparatus |
|  | endoplasmic reticulum |
|  | cytoplasmic vesicle |
|  | coated pit |
|  | extracellular region |
|  | integral to membrane |
|  | integral to plasma membrane |
|  | membrane |


---

|  |  |
| --- | --- |
| GO ID | GO:0008542 |
|  | GO:0016020 |
|  | GO:0005783 |
|  | GO:0006878 |
|  | GO:0031410 |
|  | GO:0005506 |
|  | GO:0016504 |
|  | GO:0005887 |
|  | GO:0005515 |
|  | GO:0005507 |
|  | GO:0005576 |
|  | GO:0008201 |
|  | GO:0006917 |
|  | GO:0016021 |
|  | GO:0006897 |
|  | GO:0004866 |
|  | GO:0030424 |
|  | GO:0007165 |
|  | GO:0008088 |
|  | GO:0004867 |
|  | GO:0008219 |
|  | GO:0005488 |
|  | GO:0006915 |
|  | GO:0007155 |
|  | GO:0046872 |
|  | GO:0030900 |
|  | GO:0005905 |
|  | GO:0005794 |
|  | GO:0030198 |
|  | GO:0007219 |


---

|  |  |
| --- | --- |
| MIM | MIM:104760 |


---

|  |  |
| --- | --- |
| Connectivity | 982 |


---

|  |  |
| --- | --- |
| Entrez ID | 54226 |
|  | 351 |
|  | 11820 |


---

|  |  |
| --- | --- |
| Agilent ID | A\_52\_P468472 |
|  | A\_14\_P118751 |
|  | A\_14\_P111078 |
|  | A\_53\_P133366 |
|  | A\_53\_P106797 |
|  | A\_24\_P314159 |
|  | A\_51\_P318104 |
|  | A\_14\_P124903 |
|  | A\_44\_P500880 |
|  | A\_23\_P6216 |
|  | A\_14\_P137015 |
|  | A\_52\_P110982 |
|  | A\_52\_P381311 |
|  | A\_14\_P138722 |


---

|  |  |
| --- | --- |
| Cellular Localization | Membrane |
|  | Golgi apparatus |
|  | Endoplasmic reticulum |
|  | Cytoplasmic vesicle |
|  | Extracellular region |
|  | Cytoplasm |
|  | Organelle |
|  | Cell projection |
|  | Plasma membrane |
|  | Cell |


---

|  |  |
| --- | --- |
| DbXref | KEGG pathway##05010##Alzheimer's disease##http://www.genome.jp/dbget-bin/show\_pathway?hsa05010+351 |
|  | KEGG pathway##05010##Alzheimer's disease##http://www.genome.jp/dbget-bin/show\_pathway?rno05010+54226 |
|  | KEGG pathway##05010##Alzheimer's disease##http://www.genome.jp/dbget-bin/show\_pathway?mmu05010+11820 |
|  | KEGG pathway##01510##Neurodegenerative Disorders##http://www.genome.jp/dbget-bin/show\_pathway?mmu01510+11820 |
|  | Reactome##109582##Hemostasis##http://www.reactome.org/cgi-bin/eventbrowser?DB=gk\_current&ID=109582 |


---

|  |  |
| --- | --- |
| Pathway | Alzheimer's Disease |
|  | Asthma |
|  | Apoptosis |
|  | Caspase Signaling |
|  | Zn xs inventory |
|  | Zn xs DIN |


---

|  |  |
| --- | --- |
| GO Process | endocytosis |
|  | extracellular matrix organization and biogenesis |
|  | apoptosis |
|  | induction of apoptosis |
|  | NOT axon cargo transport |
|  | signal transduction |
|  | copper ion homeostasis |
|  | Notch signaling pathway |
|  | cell death |
|  | axon cargo transport |
|  | forebrain development |
|  | cell adhesion |
|  | visual learning |


---

|  |  |
| --- | --- |
| UniGene | Rn.2104 |
|  | Hs.434980 |
|  | Mm.277585 |


---

|  |  |
| --- | --- |
| Affymetrix Probeset ID | 1371571\_at |
|  | 1380533\_at |
|  | 1398695\_at |
|  | 1420621\_a\_at |
|  | 1427442\_a\_at |
|  | 1438373\_at |
|  | 1438374\_x\_at |
|  | 1444705\_at |
|  | 1447347\_at |
|  | 1458525\_at |
|  | 200602\_at |
|  | 211277\_3p\_x\_at |
|  | 211277\_x\_at |
|  | 214953\_s\_at |
|  | 237571\_at |
|  | 243314\_at |
|  | 41136\_s\_at |
|  | 64309\_f\_at |
|  | 72834\_at |
|  | 79148\_at |
|  | 93063\_at |
|  | AFFX-hum\_alu\_at |
|  | g13325111\_3p\_at |
|  | g13325111\_3p\_x\_at |
|  | g4502166\_3p\_at |
|  | Hs.137324.0.A1\_3p\_at |
|  | Hs.177486.2.A1\_3p\_a\_at |
|  | hum\_alu\_at |
|  | Msa.10643.0\_s\_at |
|  | Msa.27234.0\_s\_at |
|  | Msa.27849.0\_s\_at |
|  | rc\_AA819527\_at |
|  | rc\_AA956735\_at |
|  | rc\_AI059313\_at |
|  | rc\_AI178039\_at |
|  | X07648cds\_at |
|  | X07648cds\_g\_at |
|  | x59379\_s\_at |
|  | Y00264\_at |
|  | 1371572\_at |
|  | 1395120\_at |
|  | 1397375\_at |
|  | 70660\_at |
|  | RC\_AA128553\_f\_at |
|  | RC\_H88303\_s\_at |
|  | TC39061\_at |
|  | TC39063\_at |
|  | W27334\_at |


---

|  |  |
| --- | --- |
| GO Function | copper ion binding |
|  | endopeptidase inhibitor activity |
|  | binding |
|  | protease activator activity |
|  | serine-type endopeptidase inhibitor activity |
|  | protein binding |
|  | heparin binding |
|  | iron ion binding |
|  | metal ion binding |


---

|  |  |
| --- | --- |
| Nucleotide | AK218128 |
|  | AK159718 |
|  | AK203089 |
|  | BC065523 |
|  | AK202486 |
|  | AK198568 |
|  | M34873 |
|  | M34865 |
|  | S60721 |
|  | AK159999 |
|  | AK194656 |
|  | BI559391 |
|  | AK194558 |
|  | AK161976 |
|  | AK190067 |
|  | AK197763 |
|  | S45136 |
|  | M16765 |
|  | AK199923 |
|  | M34866 |
|  | AK161854 |
|  | AF513015 |
|  | AY011334 |
|  | AK192103 |
|  | X59379 |
|  | AK218334 |
|  | Y00264 |
|  | AK216591 |
|  | AF199003 |
|  | X13466 |
|  | AK191703 |
|  | M28373 |
|  | BE907745 |
|  | M18734 |
|  | M35675 |
|  | A02759 |
|  | M15532 |
|  | AK216049 |
|  | M34871 |
|  | M34864 |
|  | X15210 |
|  | AK192320 |
|  | M34869 |
|  | AF056079 |
|  | AF282245 |
|  | NM\_201413 |
|  | AK029172 |
|  | AK076506 |
|  | M24397 |
|  | NM\_201414 |
|  | BC065529 |
|  | M37896 |
|  | NM\_000484 |
|  | AY267348 |
|  | AK194233 |
|  | BC005490 |
|  | M34868 |
|  | AK030583 |
|  | M34875 |
|  | BM172428 |
|  | S61380 |
|  | AK209458 |
|  | BC070409 |
|  | BC062082 |
|  | AB066441 |
|  | M34872 |
|  | M34878 |
|  | X06981 |
|  | M34874 |
|  | AK200960 |
|  | AK128971 |
|  | NM\_007471 |
|  | X06989 |
|  | AK200287 |
|  | M34876 |
|  | AK148439 |
|  | M18373 |
|  | U84012 |
|  | AF199005 |
|  | AK192899 |
|  | AK211859 |
|  | D10603 |
|  | S60317 |
|  | M24546 |
|  | M29270 |
|  | M34877 |
|  | L11926 |
|  | AK201130 |
|  | AK190928 |
|  | BC018937 |
|  | M34879 |
|  | M33112 |
|  | AY011354 |
|  | U82624 |
|  | NM\_019288 |
|  | M15533 |
|  | AK191711 |
|  | M34870 |
|  | AK086946 |
|  | X06982 |
|  | M34862 |
|  | AY011335 |
|  | M34867 |
|  | AK159245 |
|  | D87675 |
|  | AF199004 |
|  | AK190051 |
|  | S61383 |
|  | AK052448 |
|  | AK213784 |
|  | AK159352 |
|  | BC004369 |
|  | AK133639 |
|  | AK192938 |
|  | AY919674 |
|  | AK147543 |
|  | AK202618 |
|  | M34863 |


---

|  |  |
| --- | --- |
| Protein | BAE35547 |
|  | AAP23169 |
|  | NP\_958816 |
|  | CAA30041 |
|  | AAC63987 |
|  | BAE35313 |
|  | AAA35540 |
|  | AAH70409 |
|  | NP\_031497 |
|  | P12023 |
|  | CAA33280 |
|  | BAE34927 |
|  | AAA39929 |
|  | AAH62082 |
|  | AAA60163 |
|  | AAF20194 |
|  | NP\_062161 |
|  | CAA30042 |
|  | BAC34997 |
|  | BAA22264 |
|  | AAA74458 |
|  | AAH65523 |
|  | AAQ14327 |
|  | NP\_000475 |
|  | NP\_958817 |
|  | AAG47369 |
|  | AAA51727 |
|  | BAA01456 |
|  | AAB59502 |
|  | CAA31830 |
|  | AAC13654 |
|  | AAB41502 |
|  | AAA58727 |
|  | AAA51722 |
|  | AAG47389 |
|  | AAH65529 |
|  | AAB40919 |
|  | AAA51564 |
|  | AAB59501 |
|  | CAA68374 |
|  | AAA37139 |
|  | AAM90259 |
|  | AAB23646 |
|  | AAH05490 |
|  | AAB26263 |
|  | BAB71958 |
|  | CAA30050 |
|  | AAB26265 |
|  | AAA51726 |
|  | BAE27986 |
|  | AAB26264 |
|  | AAG47370 |
|  | AAC60601 |
|  | AAA51768 |
|  | CAA00268 |
|  | AAW82435 |
|  | BAC36369 |
|  | P05067 |
|  | AAH04369 |
|  | P08592 |


---

|  |  |
| --- | --- |
| Organism | Mammal |


---

|  |  |
| --- | --- |
| Location | chromosome 21, 21q21.2, 21q21.2 (Homo sapiens) |
|  | 21q21.3 (Homo sapiens) |
|  | 16 56.0 cM (Mus musculus) |
|  | chromosome 11, 11q11 (Rattus norvegicus) |
|  | chromosome 16, 16 56.0 cM, 16 C3-qter (Mus musculus) |


---

|  |  |
| --- | --- |
